# A framework for librarians to inform the citizenry during disasters: Reflections on the COVID-19 pandemic

**DOI:** 10.4102/jamba.v14i1.1197

**Published:** 2022-04-29

**Authors:** Collence T. Chisita, Patrick Ngulube

**Affiliations:** 1 Department of Information Systems, Faculty of Accounting and Informatics, Durban University of Technology, Durban, South Africa; 2 Department of Interdisciplinary Research and Postgraduate Studies, University of South Africa, Pretoria, South Africa

**Keywords:** COVID-19, infodemic, Southern Africa, access to information, partnerships, librarians and intergenerational divide

## Abstract

Globally, the coronavirus disease 2019 (COVID-19) has wreaked havoc on human lives and socio-economic activities at an unimaginable scale. African countries have not been spared from this debacle – as evidenced by media reports of loss of lives, lockdown, isolation and desolation coupled with loss of livelihood. Whilst the COVID-19 pandemic rages, libraries find themselves at the epicentre of an unprecedented crisis in the form of an information deluge that requires a multi-thronged approach to ensure information hygienic practices in information management. In order to fight COVID-19, librarians and related information professionals with relevant tools should aim at helping prevent COVID-19 pandemic infodemic (coroinfodeluge). This article explores how libraries and librarians can contribute to the fight against COVID-19 through waging wars in the realm of access to information amidst an avalanche of disinformation. This article analysed how librarians can be proactive in contributing to the fight against the COVID-19 pandemic through innovative strategies that ensure an informed citizenry. The study used qualitative content analysis as the study design. Documents were retrieved from trusted websites and they were coded before analysis. These documents included legal instruments, scholarly publications from accredited databases including Elsevier and Emerald. The study found out that librarians were not included in the national programmes to manage the COVID-19 pandemic, yet they possess potential to contribute to the fight against misinformation by educating citizens on information hygienic practices, for example, by directing users to credible or trustworthy sources on the pandemic. The study concluded that librarians can be useful stakeholders to the management of the COVID-19 pandemic and infodemic because they possess knowledge and skills relating to critical literacies that are needed in the 21st century. It recommends a collaborative framework that includes community leaders and strategic partners – to help librarians ensure that the citizenry is not misinformed during emergencies.

## Introduction

The Coronavirus disease 2019 (COVID-19) has left a trail of destruction through the loss of lives on a magnitude that is unimaginable in the 21st century. There is no country that has been spared from the loss of lives and livelihood, panic, anxiety and uneasiness as evidenced by the decision of the World Health Organization (WHO) on the 11th of March 2020 to declare COVID-19 as a global pandemic. The director of the WHO, Doctor Tedros Adhanom Ghebreyesus vehemently called upon governments to galvanise and scale up their emergency response mechanisms as a preventive and mitigatory measure (WHO 2020a). Chisita (2020) and Zarocostas (2020) concurred that it has become a preordained ritual that the outbreak of a pandemic triggers a cloud of confusion arising from misinformation and disinformation – resulting in panic, anxiety and uniformed decisions, all of which are detrimental to socio-economic stability. Social media has been chock-full with confusing posts that mislead readers and viewers. Such a scenario justifies the aforementioned call by the WHO urging governments to communicate with the citizenry regarding the risks; and the mitigatory and preventive measures against the COVID-19 pandemic. Libraries are strategically positioned to serve as a link between the government and the citizens in providing access to information to mitigate and prevent the spread of the COVID-19 pandemic and infodemic.

The critical role of libraries in disaster preparedness and response is quite phenomenal – as evidenced by the formation of the Library Roles in Disasters Project (LRDP) initiated by the United States’ National Library of Medicine (NLM) (Featherstone, Lyon & Ruffin 2008). The organisation evolved out of a recognition of the multiple roles that librarians have played in emergency and disaster planning, response and recovery. Featherstone et al. (2008) highlighted the following as some of the important roles of librarians in a disaster:

1. Institutional supporters: libraries as focal points for disaster preparedness and response
2. Collection managers: protecting, restoring and providing access
3. Information disseminators: the library as the key source of information for the entire community
4. Internal planners: providing documentation services and improvising to keep their libraries running
5. Community supporters: provision of internet access for evacuees
6. Government partners: working with government department by preparing reports and serving as referral points
7. Educators and trainers: equipping emergency responders in the use of information tools, evaluated software and provision of emergency reference services
8. Information community builder: ensuring that the citizenry is informed – providing accurate and reliable information.

Malizia, Hamilton, Littrell, Vargas and Olney (2012) mentioned the following as some of the activities through which libraries can contribute towards disaster preparedness and response:

1. Assisting the general public in locating information
2. Mobilising and distributing information
3. Multipurpose spaces in public libraries can be adapted for many uses
4. Wired public libraries can use Wi-Fi connections to broaden access including videoconferencing and satellite conferencing, which emergency responder groups can use to hold meetings among workers in dispersed areas
5. Remote access to some databases or the ability to borrow books, but public libraries extend many services and support to users without screening for place of residence
6. Providing training on consumer health information resources.

Featherstone et al. (2008) and Malizia et al. (2012) do concur on the role of librarians in emergencies with reference to providing community members with reliable and accurate information to inform the citizenry and working with government and other stakeholders to restore normalcy. Whilst both authors emphasise the provision of access to information through Wi-Fi, such an initiative is impeded by the digital divide in developing countries. Libraries have access to databases of users that can be used to inform the community, albeit with certain limitations. Librarians have skills of packaging and disseminating information at a community level. They can develop a communication strategy that can inform the framework you are envisaging.

Chiang and El Sony (2020) emphasised the need to avert the abrupt surge in the number of critically ill patients overwhelming the healthcare system, causing panic. They advised African countries to act quickly to flatten the epidemiological curve of COVID-19. Access to information is critical for the successful implementation of a disaster preparedness and response strategy. The role of libraries should not be underestimated, for instance, libraries made valuable contributions during disasters in countries outside Africa (Mabe & Ashley 2017). Librarians play a critical role in the disaster preparedness and response plan and hence the need to rethink how they can be incorporated in such challenging scenarios. Mabe and Ashley (2017) highlighted the strategic role of the public library as an agency of local government charged with providing access to information to residents. Furthermore, Mabe and Ashley (2017) opined that access to information during emergency management situations would entail the utilisation of the multimedia platforms to widen access to information. Libraries of all types have developed digital platforms whereby they can provide virtual library services, thus enabling users to access their collections (including subscriptions to electronic databases). Many academic libraries have established open educational resources (OERs) in the form of electronic theses and dissertations (ETDs) that are housed in digital institutional repositories and these can be useful in ensuring continuous access to scholarship for students and researchers (Chisita & Chiparausha 2019). The authors argue that ETDs have become deeply rooted in the scholarly ambience of academic institutions as key sources of knowledge. Electronic theses and dissertations require reliable and affordable internet connectivity and this calls for libraries to develop strategic partnerships with national research and education networks (NRENs) for affordable internet service provision (Chisita & Rusero 2016; Ojedokun & Lumande 2005). National research and education networks serve as dedicated internet infrastructure and service provider for the benefit of research and education (Chisita & Rusero 2016). The future trajectory of ETD envisages an open-access driven and interoperable global system for accessing and exchanging scholarly content to support learning, teaching and research (Fox, Gonçalves, McMillan, Eaton, Atkins & Kipp 2002).

Disaster preparedness and response is a core area of library and information science and its related disciplines, including archival sciences – hence the proposal to incorporate them in any strategic response to an emergency such as COVID-19. Disaster management refers to the continuous and integrated multisectoral, multidisciplinary process of planning and implementation of measures aimed at preventing or reducing the risks of disasters, mitigating the severity or consequences of disasters, emergency preparedness, a rapid and effective response to disasters and post-disaster recovery and rehabilitation (*South African Disaster Management Act*, 57 of 2002). Abulnour (2014) argued that the definition of a disaster should not be confined to destruction; it should include the overall effects on the community. In a study of community libraries Chizwina (2019) argued that libraries in Southern Africa were not prepared to deal with disasters, unlike their counterparts in developed countries. Scientific research has focused more on disaster preparedness rather than disaster management amongst libraries (Chizwina 2019).

Bertot et al. (2006) described library services as ‘wholly unique and immeasurably important’. According to the authors, during the time of a disaster, libraries’ provision of access to e-resources including the internet and Wi-Fi becomes more significant as the library becomes the sole access point to the outside world. However, in developing countries in sub-Saharan Africa a need exists to find a strategy to turn the digital divide into a dividend for the benefit of those who do not have access to digital technologies.

Zach (2011) argued that the role of libraries in disaster preparedness and response also differs across communities due to the perceptions and knowledge of the community members. The author attributed the degrees of differences to the different views and perceptions of the communities as they might or might not recognise libraries as key preparedness locations. This scenario is compounded by the fact that libraries are not consistently considered or acknowledged as part of a community’s disaster response matrix (Young 2018:31). Grace and Sen (2009:10) conducted research on how the British public libraries build community resilience against the challenges emanating from climate change.

According to Rani (2020), the novel COVID-19 is traced to Wuhan, China since the beginning of December 2019. However, the origins of COVID-19 remained clouded in controversy as experts, governments and citizens expressed different views. Conforti et al. (2020) viewed the COVID-19 pandemic as the most serious and dramatic public health emergency that the world has encountered in the last decades. The aforementioned authors acknowledge that despite a growing corpus of literature on COVID-19, numerous controversies still persevere – both amongst healthcare professionals and common people. The aforementioned authors opined that scientific studies dispel the myth concerning the origin of the outbreak of COVID-19 by attributing it to the laboratory experiments deliberately unleashed to cause a global crisis (Zhou et al. 2020). Cowper (2020) argued that the processes and procedures for handling the COVID-19 epidemic requires an equilibrated approach that promptly informs the citizenry on what they and the health system can do – without causing panic. Young (2018) observed that even though libraries serve as major sources of information during disasters, untimely or incomplete information is likely to cause additional confusion, chaos and anti-gregarious attitudes amongst citizens during and after an emergency.

In the United States of America, the Public Library Association (PLA) conducted a survey on how libraries were responding to the COVID-19 pandemic (American Libraries Association [ALA] 2020). The findings revealed that even though public libraries closed physically, their staff continued to serve their communities in innovative ways through utilising digital technologies, virtual programmes, communicating through phones to those in remote locations, deploying 3D printers for printing face shields, coordinating local services and information to help the homeless, utilising social media to disseminate information about the COVID-19 pandemic and switching to online programmes (ALA 2020). South Africa declared a National State of Disaster in terms of the *Disaster Management Act* 2002 on the 15th of March 2020. Zimbabwe followed the suit. The COVID-19 pandemic was declared a national disaster and mitigatory and preventive measures were announced by President Mnangagwa on the 17th of March 2020 (Mugabe 2020).

Abrams and Greenhawt (2020) observed that in the wake of disaster such as the COVID-19 pandemic whereby circumstances are in a state of constant flux and information rapidly evolving, hence the need for risk communication in order to enable access to reliable and up-to-date information for effective risk communication. The WHO (2021) defined risk communication, as ‘the exchange of real-time information, advice and opinions between experts and people facing threats to their health, economic or social well-being’. Smith (2006) distinguished between two broad risk models, namely the realist approach and the socio-cultural context. The former refers to risk as an objective and independent of social context, whilst the latter views risk from a constructivist approach whereby the phenomenon is seen to be interconnected with sociocultural context.

Hooker and Leask (2020) argued that risk communication should connect as much as possible with people’s sense of self-determination, especially in contexts where individual liberties may become limited. According to the authors the principles of risk communication imply that communication must be done early and often, being open and transparent and not dismissing concerns as ‘panic’.

Muhren and Van de Walle (2010) argued that emergencies are branded by countless types of information problems that complicate responses, for example, information disorder. This is typical of the current COVID-19 pandemic as evidenced by the proliferation of fake news and conspiracies. The COVID-19 pandemic presents librarians to rethink and restrategise on how their roles can be utilised in a scenario of emergency that grips South Africa.

## Statement of the problem

The goal of perfect epistemic experiences and a COVID-19 free world appears to be a far-fetched dream despite scientific evidence of success against a series of outbreaks of pandemics in earlier historical epochs, for example, the Spanish flu (1919–1920), Hemagglutinin 1 Neuraminidase 1 (H1N1) in 2009 and the Ebola in 2014 (Huremovic 2019). However, even though the future might appear bleak, the pundits should hold their horses in rushing to declare doomsday because scientists are working day and night to find an antidote and countries have implemented strategic measures to contain the spread of the COVID-19 pandemic. Zarocostas (2009) hinted that the world was fighting a pandemic and an infodemic that populates social networks, social media and other platforms for communication because misinformation is the greatest challenge that governments have to grapple with as COVID-19 takes its toll on human livelihoods (Chisita 2020).

The united efforts of everyone fighting COVID-19 are underpinned by access to information and knowledge that can add to perfect epistemic experiences, thus reducing uncertainty and restoring hope for humanity. The *Disaster Management Act* of South Africa (2002) does not mention the role of librarians or information professionals, yet libraries and librarians and their related institutions cannot be ignored in the information crusade against COVID-19. The librarians are key players in the COVID-19 matrix and by using technology they can leverage their knowledge and skills to educate citizens on how to overcome the information deluge (COVID-infodeluge) that continues to cause confusion, thus placing lives in danger.

## Research design

The study employed a qualitative research design using document analysis. This involved the review of documents in relationship to the topic. Dissecting documents involves coding content into subjects such as how focus group or interview transcripts are investigated. This involved the review of documents by the researchers to answer the research questions. Mackieson, Shlonsky and Connolly (2019) argued whilst document analysis has often been used in combination with other research methods as a means of triangulation to supplement and corroborate findings across different data sets with a view to minimising the impact of the potential biases in a study, it can be used as a stand-alone qualitative research method. Bowen (2009) described document analysis as a systematic procedure for reviewing or evaluating documents in order to make sense of and synthesise data contained in print and electronic documents. The researchers examined and interpreted documents relating to the COVID-19 pandemic and how libraries have responded in tackling the disaster. These were coded according to the themes of the study derived from the research questions. Bowen (2009) contended that documents can provide background information and broad coverage of data and are therefore useful in contextualising one within its subject or field.

## Purpose of the study

This study aimed to examine how libraries and librarians can contribute to the fight against the COVID-19 pandemic and infodemic in selected countries in Southern Africa with a view to developing a framework that would enable them to inform the citizenry in cases of emergency.

## Theoretical framework

Nkondo et al. (2014) proposed ‘the library and information services (LIS) transformation charter’ for South Africa with the aim of building a robust and integrated library service that facilitates the pooling and mobilisation of scarce resources. Shin (2007) observed that the National Information Infrastructure (NII) constitutes a social and technical construct because it is embedded in other structures, social arrangements and technologies. According to the author, NIIs are in a state of constant flux as they are positively and negatively affected by their sociocultural and technological milieu intricacy. Building an effective NII has become a high priority to governments around the world. Chisita and Fombad (2020) viewed the NII as a system consisting of the information environment that facilitates easier and affordable communication through connections using information and communication technologies (ICTs). The authors also describe an NII as a subset of the global information infrastructure (GII). The African Internet Government Forum (AfiGF 2015) recommended that African governments develop robust information infrastructure for an informed citizenry. African governments highlighted public access to information and knowledge to enable the realisation of Sustainable Development Goals (SDGs) as indispensable (AfIGF 2015).

Nkondo et al. (2014) viewed an ecosystem as a system consisting of subsystems that are interlinked, interactive and interdependent within the context of co-evolution whereby change is constant (Nardi & O’Day 1999). Walter (2008) described the library as an ecosystem that encompasses the interactions between the library and its internal and external publics. Furthermore, Walter (2008) considered the library as a habitat for multiple species and an institution whose relations continue to grow through space and time. The concept of ecosystems is derived from ecology, which is a discipline that studies the interactions between organisations and their environments. Mars, Bronstein and Lusch (2012) used the metaphor of ecosystems to represent the interface amongst organisations that share common features and that justify or facilitate some form of exchange of information and other resources. According to the author, ecosystems refer to social structures that comprised loose and tight ties that enable or enhance the interactions amongst diverse organisations and actors.

García-Marco (2011) considered libraries to be the main bridges between available knowledge and knowledge gaps. According to the author, libraries are a constituency of a huge group of industries that cooperate and compete in permanently providing information to users and in developing a series of activities to connect information demand with information supply. Libraries do not have sole preserve to transfer or share knowledge because there are alternative channels through which social knowledge circulates and a whole set of competing industries maintaining them (García-Marco 2011).

The global prevalence of misinformation has resulted in government’s reactivation of their media control institutions and the invocation and enactment of new laws to deal with the misinformation crisis amidst the COVID-19 pandemic. Hua and Shaw (2020) pointed out that access to correct and timely information is key to fighting the COVID-19 pandemic and infodemic. The era of information disorder arising from fake news can lead to a scenario whereby the world will rapidly be influenced by self-proclaimed experts from the opinion market (Cook, Lewandowsky & Ecker 2017).

The term infodemic refers to the perils of misinformation phenomena during the management of virus outbreaks because it could even speed up the epidemic process by influencing and fragmenting social response (WHO 2020b Novel Coronavirus (2019-nCoV) Situation Report - 13; Zarocostas 2020). Cinelli et al. (2020) observed that the onus is on researchers to determine how people seek or avoid information and how those decisions affect their behaviour, especially when the news cycle – dominated by the disintermediated diffusion of information – alters the way information is consumed and reported on. There is growing concern over the phenomenal growth of misinformation in society, arising from unedited information sourced from crowds (Qutab, Myers & Gardner 2019). Chisita et al. (2020) raised concern on how content from unprofessional contributors ends up shaping public opinion and breeding conspiracy theories. There is a growing concern over the misuse of social media platforms to peddle fake news, resulting in information disorder. Libraries, archives and the media can help to equip users and readers with knowledge and skills to overcome information disorder. Floridi (2013:15) regarded the philosophy of information as a discipline primarily concerned with ‘how information should be adequately created, processed, managed and used’ (p. 15). Inaccurate and misleading information can be extremely dangerous. Fallis (2015) opined that disinformation can cause significant harm if people are misled by it. Disinformation is described as misleading information that has the function of misleading (Fallis 2015). The author warns libraries and related information services against the danger of intentionally and unintentionally becoming conduits for the spread of disinformation. Through the use of a formal model, Fallis (2014) showed that the amount of disinformation tends to decrease when:

1. benefits to the recipient, of believing what the source says when it is true, decrease
2. costs of believing what the source says when it is false, increase
3. chances that the source has a motivation to deceive, increase.

The given assumptions can be useful in devising policies and frameworks that might deter people from disseminating disinformation. Such information is conveyed through social media platforms, as highlighted in the previous sections. Mutsvairo and Rønning (2020) described social media as Janus faced, implying that it reflects a double-edged characteristic, for example, it acts as conveyor of ideologies that misrepresent reality and on the contrary it also serves to challenge asymmetric power structures of domination and exploitation. Social media can also be a platform for antisocial communication. The misuse of social media has resulted in governments promulgating laws to ensure sanity amidst the menacing COVID-19 pandemic and infodemic. Whilst the new COVID-19 legislation and regulations might sound draconian, it should be observed that in times of emergencies governments usually have no option but to implement such measures as long as they will benefit the nation.

South Africa’s Regulations issued in terms of Section 27(2)0 of the *Disaster Management Act* 2002, section 11, subsection 5 stipulate that:

[*A*]ny person who intentionally misrepresents that he, she or any other person is infected with COVID-19 is guilty of an offence and on conviction liable to a fine or to imprisonment for a period not exceeding six months or to both such fine and imprisonment … Any person who publishes any statement, through any medium, including social media, with the intention to deceive any other person about – (1) COVID-19; (2) COVID-19 infection status of any person; or (3) any measure taken by the Government to address COVID-19, commits an offence and is liable on conviction to a fine or imprisonment for a period not exceeding six months, or both such fine and imprisonment.

The government of Botswana promulgated the Statutory Instrument No. 61 of 2020 *Emergency Powers Act* (cap. 22:04) Emergency Powers (COVID-19) Regulations, 2020 whereby Section 30 subsection 1 states that:

[*A*]ny person who relays any information to the public about COVID-19 from a source other than the Director of Health Services, and the WHO commits an offence and is liable to a fine not exceeding P100 000 or to imprisonment for a term not exceeding 5 years, or to both… (3) A person who publishes any statement, through any medium, including social media, with the intention to deceive any other person about….

Zimbabwe gazetted the Statutory Instrument (SI) 83 of 2020. [CAP. 15:17 Public Health (COVID-19 Prevention, Containment and Treatment) (National Lockdown) Order, 2020 criminalises the publication and communication of fake news relating to the COVID-19 pandemic (Machivenyika 2020). Madhuku (2020), a constitutional law expert, cited by Mugabe, in the *Herald* (17 March, 2020) hinted that the promulgation of SI was constitutional. Globally, countries that have declared lockdown have had to promulgate laws to justify their actions:

[… *A*]ny person who publishes or communicates false news about any public officer, official or enforcement officer involved with enforcing or implementing the national lockdown in his or her capacity as such, or about any private individual that has the effect of prejudicing the State’s enforcement of the national lockdown, shall be liable for prosecution …and liable to the penalty there provided, that is to say a fine up to or exceeding level fourteen or imprisonment for a period not exceeding twenty years or both. (Statutory Instrument [SI] 83 of 2020. (CAP. 15:17 Public Health [COVID-19 Prevention, Containment and Treatment] [National Lockdown] Order 2020)

Hodgson, Kanyo and Mavedzenge (2020) observed that Southern African governments in their endeavour to mitigate and prevent the spread of COVID-19 pandemic had raced to enact regulations providing for the restrictions of certain fundamental rights and freedoms, for example, freedom of movement and association. The regulations against fake news are the most notable laws meant to help in mitigating and preventing COVID-19 in the region. Head (2020) reported the case of a man in South Africa who was arrested for attempting to spread fear and panic directly, thus contriving new regulations passed during the lockdown period from 27 March to 30 April 2020. The arrest was triggered by a video that was disseminated through various social media platforms, purporting contamination of COVID-19 test kits. The peddling of fake news contravenes the South African *Disaster Management Act* 57 of 2002.

The National Information Authority Uganda (Ntezza 2020) issued an advisory on common scams the public should look out for as they embrace social distancing and working from home during the nationwide lockdown. The advisory warned the citizenry to be on the lookout for scams and scenarios that can lead to cybercrime exposure, for example, fake news and rumours:

[*W*]e know that every outbreak will be accompanied by a kind of tsunami of information, but also within this information you always have misinformation, rumours, etc … But the difference now with social media is that this phenomenon is amplified, it goes faster and further, like the viruses that travel with people and go faster and further. So it is a new challenge, and the challenge is the [timing] because you need to be faster if you want to fill the void … What is at stake during an outbreak is making sure people will do the right thing to control the disease or to mitigate its impact. So it is not only information to make sure people are informed; it is also making sure people are informed to act appropriately. Briand (2020)

There is a need for a framework that spells out the role of the library in times of emergency with reference to COVID-19. The following figure illustrates how libraries can fit in the emergency matrix in case of a disaster.

The following section will describe and explain how the given framework works.

### United Nations

The United Nations Charter (UN) (1945) Chapter 1, article 1, aims to:

[*A*]chieve international co-operation in solving international problems of an economic, social, cultural, or humanitarian character, and in promoting and encouraging respect for human rights and for fundamental freedoms for all without distinction as to race, sex, language, or religion.

Solidarity and coordinated action is key to the fight against the COVID-19 pandemic:

[*T*]he UN is responding with determination and coordination, bringing the UN entities together to support governments, while also continuing our urgent response to humanitarian coordination, working for peace and prosperity for all. (António Guterres 2020)

United Nations teams across the globe are working closely with governments, agencies, partners and civil society to ensure the safety, security and health of the population. The United Nations Interdepartmental Framework for Coordination Team aims to support preventive action and to develop improved mechanisms for early warning, contingency planning and preparedness.

### World Health Organization

Ruger and Yach (2009) argued that the global health landscape in the 21st century requires effective global action in the face of globalisation of trade, travel, information, human rights, ideas and disease. Furthermore, the authors highlight the importance of coordination of effort, priorities and investments to combat global health challenges. The WHO is responsible for universal health governance through working with member states of the UN. During the current COVID-19 pandemic and infodemic it has been collating country reports, monitoring and evaluating mitigation and prevention measures, sharing information and knowledge and providing guidelines and regulations for mitigatory and preventive measures against COVID-19 (WHO 2020b).

### Government of the Republic of South Africa

The South African government’s *Disaster Management Act* (DMA 2002) and the National Disaster Management Framework (NDMF) constitute an integral policy providing the legislative and regulatory support to deal with emergencies (DMA 2002). The *Disaster Management Act* of South Africa (2002) empowers every government entity (national, provincial and local) to play an active role in mitigating and preventing disasters (Van Niekerk 2005). It was the government who declared COVID-19 a national disaster and placed South Africa under lockdown for 21 days from the 27th of March 2020 and then it was extended for 2 weeks to the 16th of April, 2020. The lockdown continued to be monitored and subjected to reviews depending on the situation on the ground (South African Government 2022). The government has produced and communicated regulations and guidelines for road and air travel, spaza shops, social functions including funerals and weddings, exemptions for essential services, ICTs and essentials for sale during the lockdown.

### National Disaster Management Centre

According to the DMA (National Disaster Management Centre) (2020), the National Disaster Management Centre is established in terms of Section 8 of the *Disaster Management Act*, 2002 (Act 57 of 2002). Its main objective is to promote an integrated and coordinated system of disaster management, with special emphasis on prevention and mitigation, by national, provincial and municipal organs of state, statutory functionaries, other role-players involved in disaster management and communities. This institution has managed to work with stakeholders including those outlined in the collaborative framework for libraries in times of emergency (COVID-19 pandemic). The National Disaster Management Centre is driven by the philosophy that combating a national disaster is a shared responsibility as highlighted in the proposed framework of this study. It serves as a source of reliable and accurate information to support decision-making. Its linkages to all types of libraries has been useful in ensuring access to reliable information on the COVID-19 pandemic.

### National Institute for Communicable Diseases

This institute is the national public health institute of South Africa, providing reference microbiology, virology, epidemiology, surveillance and public health research to support the government’s response to communicable disease threats (NICD 2020). The NICD serves as a resource of knowledge and expertise in the field of communicable diseases to the South African Government, Southern African Development Community (SADC) and the African continent. The Institute assists in the planning of policies and programmes to support and respond to communicable diseases (NICD 2020).

## Library ecosystem

This ecosystem consists of all the libraries in South Africa, including their internal and external systems, processes and procedures. It also includes how the libraries interact amongst themselves and with others on regional and international level. The library as an ecosystem exists in a socio-economic and political milieu because its existence is inseparable from its environment. The ecosystem metaphor implies that the library system comprises multiple species that interact with each other and their environment and includes the interaction amongst library professionals and between library professionals and other stakeholders, including users (Walter 2008). The library as a system, nourished and sustained by multiple interactions, is vulnerable to the disturbances in the environment. The current COVID-19 pandemic and infodemic have forced libraries that are resource starved to close, whilst those endowed with technological resources continue to provide virtual services. Walter (2008) argued that libraries as biological organisms evolve in order to adjust to time. The Gauteng Department of Sports, Arts, Culture and Recreation (GDSACR) has opened its virtual library to residents, including those with no membership cards (Hlophe 2020). Whilst most libraries closed their services prior to the lockdown (thus, before the 18th of March), they continue to offer virtual support – thus allowing users to navigate databases, COVID-19 resources and referencing services amongst others. For instance, open distance e-learning (ODeL) institutions continue to support their students and researchers through virtual library services.

## World Health Organization regional and country representatives

The member states of the WHO are organised according to six regions consisting of Africa, South East Asia, Eastern Mediterranean, European and Western Pacific. Each member state has a country representative who is the link person between the host government and the WHO. World Health Organization regional offices strengthen the organisation’s country presence by developing and implementing regional cooperation strategies supporting country teams in the implementation of the country cooperation strategy.

The WHO country offices play an important role within the organisation in ensuring appropriate WHO technical cooperation with countries and leadership in the health sector. The country offices have three main functions: policy advice and technical support; information, public relations and advocacy; and management and administration. Libraries of all types can access the WHO website for information to cascade to scholars, researchers and the community on the COVID-19 pandemic. Academic and research libraries have utilised their digital networks to access COVID-19 pandemic resources to support learning, teaching and research.

## Strategic local, regional and global partners

These partnerships are useful as they enable inter-institutional cooperation and collaboration through mobilising resources in order to tackle the COVID-19 pandemic. The NICD has partnerships with local and international organisations, for example, the World Health Organization, Centres for Disease Control and Prevention, Africa Centres for Disease Control and Prevention, European Centres for Disease Control and Prevention, Public Health England, as well as many other credible organisations in the public health space to support activities and efforts towards the improvement of public health. Partnerships are important in the fight against the COVID-19 pandemic because they provide a platform to share best practices and facilitate knowledge exchange, surveillance and disease intelligence and resource mobilisation. These partnerships have enabled academic and research libraries to access open resources and other scholarly communication to support their mission as centres of knowledge.

## Internet service providers

Access to the internet during the COVID-19 era has become a topical issue across the globe as governments grapple with the challenge of how to ensure that people can continue their studies in the comfort of their homes. Internet service providers (ISPs) provide internet connectivity and can thus provide a solution to the connectivity problem during times of crisis. The ISPs consist of commercial and non-commercial ISPs. The non-commercial ISPs are referred to as NRENs. They serve as dedicated internet infrastructure and service providers for the benefit of research and academic institutions (Chisita & Rusero 2016). The secondary role of NRENs is to extend its service to communities of interest (COIs) and communities of practices (COPs), government institutions and other key stakeholders (Chisita & Rusero 2016). Zennaro et al. (2020) pointed out that NRENs were useful in meeting the specific needs in terms of bandwidth, quality of service, security and reliability of the research and education communities. Furthermore, the authors note that such needs cannot typically be met by commercial providers because they would require high costs with possibly low-economical returns.

Internet service providers are key pillars of the NII constituted of people, communication networks, ICT hardware and software and information and knowledge resources (Chisita 2020). Currently, the South African National Research Network (SANReN) and the Tertiary Education and Research Network (TENET) are providing resources to move large data in a secure environment and availing resources for research and data analysis relating to efforts to mitigate and prevent the COVID-19 pandemic (SANReN 2020). These institutions are useful in providing internet and Information Technology services to academic institutions. The community of NRENs includes universities, research and development institutions, primary and secondary schools, libraries, museums, hospitals, telecom service providers and internet exchange points (IXPs) (Zennaro et al. 2020). The Internet Service Providers Association of South Africa (ISPA) issued a statement on the need South African ISPs and other ICT service providers to take steps to support internet users engaged in online education during the COVID-19 National Disaster period. These steps may include zero-rating educational traffic, temporarily increasing bandwidth caps for some customers or even providing limited free ‘lifeline’ data packages to customers (ISPA 2021). This imitative has been beneficial to digital libraries involved in supporting learning, teaching and research during the COVID-19 era.

## Communities

MacQueen et al. (2001) defined a community as a group of people with diverse characteristics who are linked by social ties, share common perspectives and engage in joint action in geographical locations or settings. This study adopts a definition of a community as referring to a group of people whose social interaction is intertwined by cooperation and collaboration to avert and convert the COVID-19 pandemic within a specific geographical location in order to restore life to normalcy. The community comprises the people of South Africa and their values, institutions and beliefs. The community is constituted of citizens, leaders, community leaders, opinion leaders, social groups, religious-political and other social entities. These social entities have diverse information needs that the library as an ecosystem can respond to. However, the challenge, that confronts libraries and other stakeholders relates to the capacity to provide for information needs of users amidst the digital divide, hence the need for partnerships with government, ISPs and local, regional and international organisations involved in the fight against the COVID-19 pandemic.

## How can the knowledge and skills of librarians be useful in an emergency?

**FIGURE 1:**
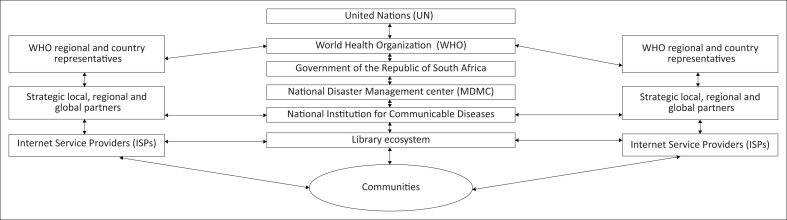
Collaborative framework for libraries in times of emergency (COVID-19 pandemic).

Wang and Lund (2020) observed that whilst governments have an overall responsibility to implement initiatives to mitigate and prevent the spread of the COVID-19 pandemic such as, health and safety, travel and transportation, business and education, libraries have an equal role in providing emergency services as highlighted in the given framework. Featherstone et al. (2008) highlighted that libraries can serve as a ‘command centre for activities’, to support members of the community by ensuring the maintenance of the collection throughout the disaster, disseminating reliable information about ongoing events, providing support to staff, providing emotional support and distributing donations, partnering with the government to develop/distribute reports on an evolving situation, educating and training the community on the disaster, curating information and providing instruction for emergency responders and information community building through restoring the normal functioning of the library during and after the disaster. The given ‘collaborative framework for libraries in times of emergency (COVID-19 pandemic)’ shows how libraries as ecosystems are interlinked with the community, government, ISPs, regional and international organisations. The linkages in the framework help to ease information and communication flows on the pandemic and they also ensure normal functions for example, if ISPs subsidise data it becomes easier to providing digital library services as is the current trend in South Africa’s academic institutions. Wang and Lund (2020) contended that libraries play a vital role in providing reliable information about pandemics including COVID-19, using their strengths in gathering, evaluating and curating information for the public. The authors stated that librarians should not downplay their unique skills and the important role they may play in curbing the spread/impact of the COVID-19 pandemic and infodemic. However, libraries can only manage to do more provided they are cooperating and collaborating with other stakeholders as highlighted in Figure 1.

## Conclusion

The collaborative framework for librarians in the wake of the COVID-19 pandemic (Figure 1) positions librarians as stakeholders in the fight against the pandemic. The library ecosystem represents librarians as an interlinked network of information providers. Librarians interact with all stakeholders including, commercial and non-commercial ISPs, communities, local and regional organisations, government and the World Health Organisation (WHO). Despite that throughout the region librarians were not explicitly mentioned as critical in the mitigation and prevention plans, they were able to switch on their digital platforms to provide remote access in support of learning, teaching and research. Furthermore, their usefulness has been evidenced by their ability to provide active links to trusted sources of information relating to the COVID-19 pandemic. The proposed framework for librarians in the wake of the COVID-19 pandemic and infodemic recognises and values the skills and knowledge of librarians in dealing with such challenges as the infodemic. Such misinformation emanates from formal and informal channels of communication, including the private sector and the public sector and development agencies. This scenario is compounded by the avalanche of information that Chisita and Ezema (2020) referred to as the coroinfodeluge. The coroinfodeluge refers to the avalanche of COVID-2019 information that proliferates social media, thus overwhelming the capacities of individuals to analyse and make judicious use of the information (Chisita & Chisita 2021). As conduits to trustworthy information, libraries should play a role in providing access to information for the citizens. Librarians contribute to an informed citizenry by empowering users with metaliteracy skills that emphasise the importance of critical thinking skills. Metaliteracy has been signalled as the best-fit for nurturing a culture of reflective learning, active and critical immersion with regards to the COVID-19 pandemic’s abundant grounds of misinformation and disinformation. On a similar note (Parvin, 2019) disasters bring uncertainty and despondence amongst citizens because of the proliferation of untrustworthy sources or platform on the internet and librarians can leverage their social capital, linkages and expertise to promote information hygienic use of COVID-19 pandemic information. It is out of this context that the researchers recommend that librarians to be considered as key contributors in national disaster management teams in the fight against the COVID-19 pandemic. The proposed framework (Figure 1) views librarians and libraries as part of NII, which represent a subcategory of the GII (AfIGF 2015).

The library ecosystem should collaborate with local and international stakeholders in order to facilitate access to credible sources of information and enable users to make sense of the avalanche of COVID-19 pandemic information that proliferates the internet. Access to information may make a huge difference to the way citizens respond to the risk and spread of the pandemic, hence the need for information management for effective risk communication as highlighted and re-emphasised by WHO (2021), Smith (2006) and the proposed framework.
